# Traumatic Bilateral Asymmetrical Hip Dislocation with Acetabular Fracture: A Case Report and Review of Current Evidence

**DOI:** 10.3390/life15040532

**Published:** 2025-03-24

**Authors:** Jovana Grupkovic, Uros Dabetic, Nikola Bogosavljevic, Dejan Aleksandric, Mladen Milanovic, Dunja Savicevic, Slavisa Zagorac

**Affiliations:** 1 Clinic for Orthopedic Surgery and Traumatology, University Clinical Center of Serbia, 11000 Belgrade, Serbia; urosdabetic1983@gmail.com (U.D.); mladenmilanovic462@gmail.com (M.M.); slavisa.zagorac@gmail.com (S.Z.); 2 Faculty of Medicine, University of Belgrade, 11000 Belgrade, Serbia; boga19@gmail.com; 3 Institute for Orthopedic Surgery “Banjica”, 11000 Belgrade, Serbia; aleksandricdejan@gmail.com; 4 Special Hospital for Rehabilitation and Orthopedic Prosthetics, 11000 Belgrade, Serbia; savicevic.dunja@gmail.com

**Keywords:** bilateral hip dislocation, asymmetrical hip dislocation, acetabular fracture, orthopedic trauma, avascular necrosis, post-traumatic osteoarthritis, hip rehabilitation

## Abstract

Bilateral asymmetrical hip dislocations are rare, occurring in only 0.01–0.02% of all joint dislocations, typically following high-energy trauma. We present a 22-year-old male involved in a high-speed motor vehicle collision, sustaining a right posterior hip dislocation with an associated posterior wall acetabular fracture and a left obturator-type anterior dislocation. He underwent successful closed reduction within two hours post-injury, but due to persistent instability of the posterior acetabular wall fracture, open reduction and internal fixation (ORIF) via a Kocher–Langenbeck approach was performed. A structured rehabilitation protocol facilitated full functional recovery at six months, with no evidence of avascular necrosis (AVN) or post-traumatic osteoarthritis. A literature review of relevant studies highlights the importance of early reduction (<6 h) to reduce AVN risk, timely surgical stabilization for acetabular fractures, and individualized rehabilitation strategies. While our case supports established treatment guidelines, long-term outcomes and optimal rehabilitation protocols remain areas for further research. Expedited diagnosis, early intervention, and evidence-based management are essential in achieving favorable outcomes for these complex injuries.

## 1. Introduction

Traumatic hip dislocations are rare but severe injuries, typically resulting from high-energy mechanisms such as motor vehicle collisions or falls from significant heights. Posterior dislocations account for approximately 85–90% of cases, while anterior dislocations comprise 10–15% [1]. Bilateral hip dislocations are exceedingly uncommon, with an incidence of approximately 1% of all hip dislocations, and asymmetrical bilateral dislocations represent an even rarer subset, occurring in only 0.01–0.02% of all joint dislocations [2,3]. Due to their infrequency, these injuries pose significant diagnostic and therapeutic challenges, necessitating a thorough evaluation to identify and manage potential associated fractures or neurovascular compromise.

Management of traumatic hip dislocations must be prompt to mitigate complications such as avascular necrosis of the femoral head and post-traumatic osteoarthritis [4]. Early closed reduction is the standard of care when feasible, but cases involving associated acetabular fractures often require surgical intervention [5]. The optimal treatment approach remains a subject of debate, with literature advocating for early surgical fixation in cases of unstable reductions, large acetabular wall defects, or intra-articular loose bodies [4].

Due to this extreme rarity mentioned above, most of the available literature consists of isolated case reports and small case series, leading to a lack of standardized treatment protocols and long-term outcome data. While early reduction and surgical fixation are widely accepted principles, optimal timing for surgery, post-reduction stability assessment, and structured rehabilitation strategies remain poorly defined. Additionally, the risk factors for avascular necrosis (AVN) and post-traumatic osteoarthritis in these cases are not well established, complicating long-term prognostic evaluations. This narrative review and case report aim to fill this gap by synthesizing the existing case-based evidence, identifying key trends in injury mechanisms, management strategies, and clinical outcomes, and proposing evidence-based considerations for future research and treatment guidelines.

Building on this, we present a complex case of bilateral asymmetrical traumatic hip dislocation with an associated posterior wall acetabular fracture, highlighting the mechanisms of injury, diagnostic protocols, management strategies, and patient outcomes. Additionally, a review of the current literature is provided to contextualize this case within the broader spectrum of traumatic hip dislocations and to discuss best practices in evaluation, surgical decision making, and rehabilitation.

## 2. Case Presentation

A 22-year-old male was involved in a high-speed motor vehicle collision as a restrained front-seat passenger. Upon arrival at our Level I trauma center one hour post-injury, he was hemodynamically stable but reported severe pelvic and bilateral hip pain, complete inability to bear weight, and restricted active hip motion. Primary survey per Advanced Trauma Life Support (ATLS) guidelines identified no immediate life-threatening injuries. Secondary examination revealed asymmetrical lower limb positioning, with the right lower extremity fixed in flexion, adduction, and internal rotation, while the left lower extremity was held in flexion, abduction, and external rotation, consistent with a right posterior hip dislocation and left anterior-inferior hip dislocation.

A focused neurovascular assessment demonstrated preserved distal pulses bilaterally, with no clinical evidence of sciatic or femoral nerve injury. Capillary refill was within normal limits, and motor function testing of the quadriceps, tibialis anterior, gastrocnemius, and extensor hallucis longus was intact. There were no signs of open wounds, external bleeding, or pelvic instability.

**Figure 1:**
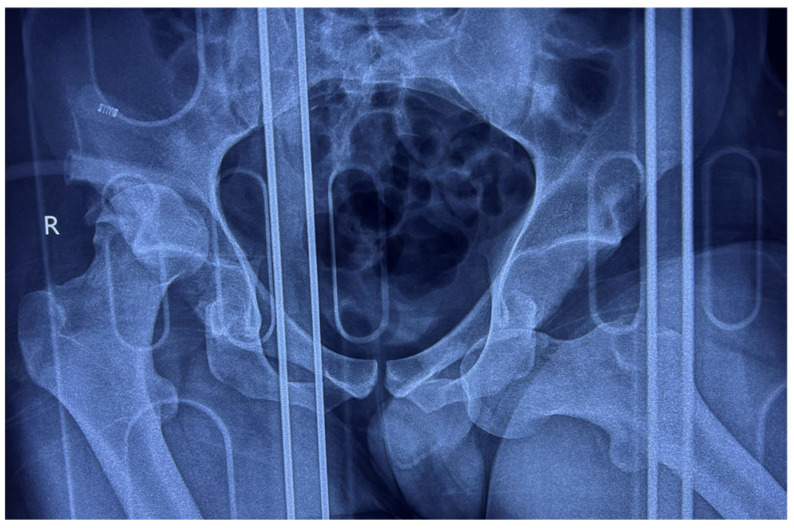
AP radiograph of the pelvis showing bilateral asymmetric hip dislocation with right femoral head displaced posteriorly beyond the acetabular rim, with an associated acetabular posterior wall fracture, while the left femoral head is displaced anteroinferiorly into the obturator foramen.

Anteroposterior (AP) pelvic radiographs immediately confirmed the presence of a bilateral asymmetric hip dislocation (Figure 1). The right femoral head was displaced posteriorly beyond the acetabular rim, with an associated acetabular posterior wall fracture, while the left femoral head was displaced anteroinferiorly into the obturator foramen, indicating an obturator-type anterior dislocation.

**Figure 2:**
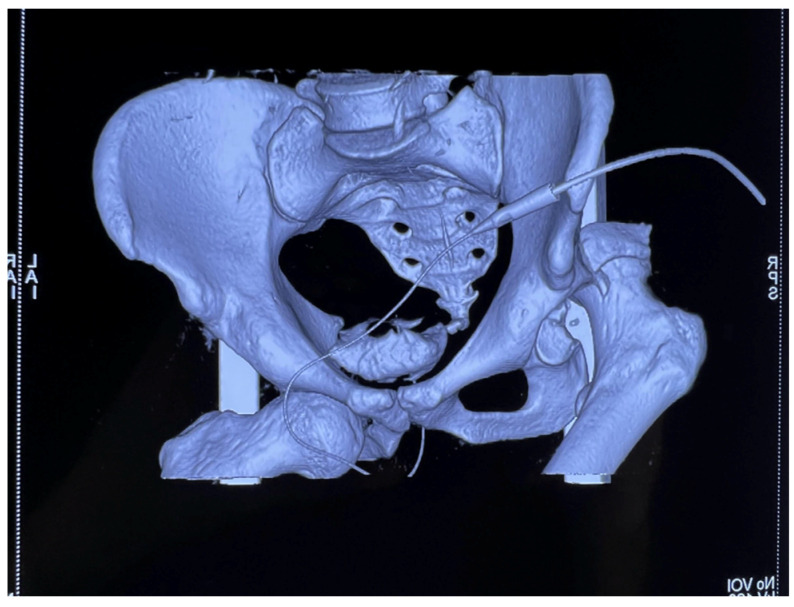
MSCT of the pelvis with 3D reconstruction showing right posterior wall acetabular fracture and confirming there are no femoral head fractures, intra-articular fragments, marginal impaction, nor pelvic ring disruptions.

Subsequent multislice computed tomography (MSCT) of the pelvis with 3D reconstruction provided enhanced visualization of the osseous and articular morphology (Figure 2), confirming the following:

- Right posterior wall acetabular fracture, with an estimated >25% involvement of the weight-bearing dome, necessitating surgical intervention.
- No femoral head fractures, intra-articular fragments, or marginal impaction.
- No sacroiliac joint disruption or pelvic ring instability.

**Figure 3:**
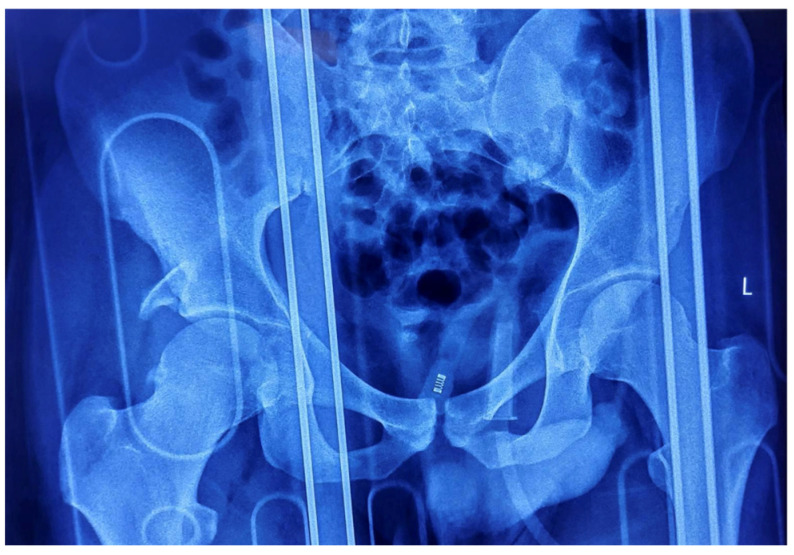
Post-reduction AP radiograph of the pelvis showing joint congruity, as well as the right posterior acetabular wall fracture.

The patient underwent closed reduction under general anesthesia within two hours post-injury using fluoroscopic guidance. The Allis maneuver was employed for the right posterior dislocation, and the Captain Morgan maneuver was utilized for the left anterior dislocation, achieving concentric reduction on both sides. Post-reduction radiography confirmed joint congruity, but the right posterior acetabular wall fracture remained unstable, necessitating operative intervention (Figure 3).

- Definitive management was performed three days post-injury via an open reduction and internal fixation (ORIF) of the right posterior acetabular wall using a Kocher–Langenbeck approach. Intraoperative findings included the following:
- A comminuted posterior wall fragment with associated capsular detachment.
- Successful anatomic reduction and fixation achieved using a 3.5 mm reconstruction plate and cortical screws, restoring acetabular integrity.
- Intraoperative dynamic testing confirmed joint stability, with no evidence of residual subluxation or impingement.

**Figure 4:**
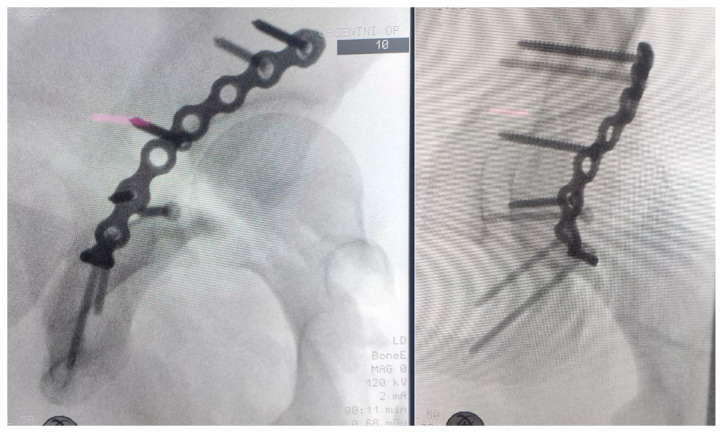
Intraoperative Judet views—iliac oblique view, and obturator oblique view of the right hip—showing anatomic reduction of posterior wall fracture and proper positioning of osteosynthetic material.

**Figure 5:**
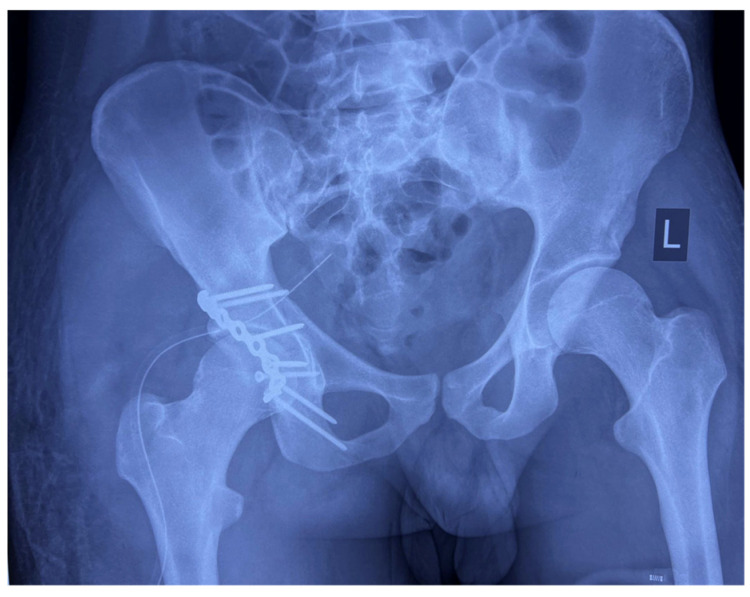
Postoperative AP radiography of the pelvis showing anatomic reduction of posterior wall fracture and proper positioning of osteosynthetic material.

Intraoperative radiographs as well as postoperative radiographs demonstrated anatomic reduction of the posterior wall fragment with proper positioning of the osteosynthetic material (Figure 4 and Figure 5). The postoperative neurovascular examination was completely normal, with no clinical signs of sciatic nerve injury.

At the first follow-up, 14 days postoperatively, the surgical wound had healed by primary intention without any complications.

Postoperatively, the patient was maintained on DVT prophylaxis with low-molecular-weight heparin (LMWH) and heterotopic ossification prophylaxis with indomethacin. He followed a structured rehabilitation protocol, initially restricted to toe-touch weight-bearing on the right lower extremity for six weeks, with progressive advancement to full weight-bearing by 12 weeks.

A structured rehabilitation program was initiated immediately postoperatively (day 0) to prevent cardiopulmonary, osteomuscular, and thromboembolic complications. Early mobilization was emphasized to optimize functional recovery while ensuring adequate pain control through multimodal analgesia. Functional progress was monitored using the Harris Hip Score (HHS), Timed Up and Go (TUG) test, and Berg Balance Scale (BBS).

### 2.1. Phase 1: Early Mobilization and Initial Weight-Bearing (Postoperative Weeks 0–6)

From the first postoperative day, the rehabilitation protocol included the following:

- Respiratory exercises to prevent pulmonary complications.
- Peripheral circulation exercises (ankle pumps, active dorsiflexion, and plantarflexion) to reduce the risk of deep vein thrombosis (DVT).
- Isotonic exercises for the upper extremities to maintain overall muscle conditioning.
- Isometric (static) strengthening exercises for the quadriceps, hamstrings, gluteal, and pelvic trochanteric musculature.
- Heel slides and active-assisted ROM exercises for the hip and knee to preserve joint mobility.
- Touch-down weight-bearing (TDWB) was permitted with the use of axillary crutches, with gradual daily progression in ambulation distance.

Objective Functional Assessment:

- HHS at Week 2: ~50 (Preserved ROM, pain well controlled, ambulation with assistive devices).
- HHS at Week 6: ~65 (Increased endurance, progressive ROM recovery, independent transfers).

Since balance and stability were crucial for ambulation, the Berg Balance Scale (BBS) was used at the end of the first phase:

- BBS at Week 6: 35/56 (Moderate balance impairment, requiring assistive support).

The TUG test was not applicable in the early stage due to restricted weight-bearing.

### 2.2. Phase 2: Progressive Weight-Bearing and Strengthening (Weeks 6–8)

From week 6 to week 8, rehabilitation advanced with the following:

- Active and active-assisted ROM exercises against gravity.
- Stationary cycling (30 min per session) to enhance endurance and joint proprioception.
- Progressive weight-bearing up to 50% of body weight, dynamically assessed based on tolerance and radiographic findings.

Objective Functional Assessment:

- HHS at Week 8: ~75 (Partial weight-bearing tolerated, improved ROM, reduced reliance on assistive devices).
- BBS at Week 8: 42/56 (Improved balance, independent standing, but still requiring gait assistance).
- TUG at Week 8: 16.2 s (Moderate improvement in mobility).

### 2.3. Phase 3: Advanced Rehabilitation and Functional Training (Weeks 8–10)

From week 8 to week 10, the patient progressed to the following:

- Weight-bearing increase to 75%, transitioning to a single forearm crutch.
- Dynamic lower extremity exercises to reinforce muscular endurance and stability.
- Balance and coordination training to prevent compensatory gait abnormalities.

Objective Functional Assessment:

- HHS at Week 10: ~85 (Near-normal gait mechanics, minimal residual discomfort).
- BBS at Week 10: 48/56 (Improved dynamic balance, minimal sway during single-limb stance).
- TUG at Week 10: 11.5 s (Nearly restored walking speed, stable pivot turns).

### 2.4. Phase 4: Return to Full Weight-Bearing and Independent Mobility (After Week 12)

By week 12, full weight-bearing as tolerated (FWBAT) was allowed, ensuring the patient demonstrated the following:

- Negative Trendelenburg sign, indicating adequate hip abductor strength.
- Independent gait pattern without assistive devices.
- Pain-free functional mobility, with no signs of post-traumatic instability.

Objective Functional Assessment:

- HHS at Week 12: ~90 (Near-full function, minimal residual limitations, independent ambulation).
- BBS at Week 12: 54/56 (Functional balance restored, full stability).
- TUG at Week 12: 9.8 s (Within normal range for age-matched controls).

### 2.5. Month Follow-Up

- HHS: 95+ (Full weight-bearing, full ROM, return to pre-injury activity level).
- BBS: 56/56 (Optimal balance, no limitations).
- TUG: 8.9 s (Equivalent to healthy population).

The stepwise approach to weight-bearing and strengthening ensured safe progression and optimal recovery, leading to full functional restoration at the six-month follow-up.

At the six-month follow-up, the patient exhibited the following:

- Pain-free, full weight-bearing ambulation without assistive devices.
- Full restoration of hip range of motion bilaterally, with no signs of post-traumatic instability.
- No radiographic evidence of avascular necrosis (AVN) or post-traumatic osteoarthritis.

This case underscores the importance of early closed reduction, timely surgical stabilization, and structured rehabilitation in optimizing outcomes for complex asymmetrical bilateral hip dislocations.

The chronological sequence of events for this case is outlined in Table 1.

## 3. Review of Literature

### 3.1. Study Design

This study was conducted as a narrative review alongside a case report to provide a comprehensive discussion on traumatic bilateral asymmetrical hip dislocations with associated acetabular fractures. Given the extreme rarity of this injury pattern, most of the available literature consists of isolated case reports and small case series, making a systematic review impractical due to the lack of high-quality observational studies.

Unlike systematic reviews, which follow PRISMA guidelines with predefined inclusion/exclusion criteria and a structured quality assessment, this narrative review focuses on qualitatively synthesizing available case reports and small case series. The aim is to identify patterns in injury mechanisms, treatment approaches, and clinical outcomes, while highlighting gaps for future research.

#### 3.1.1. Literature Search Strategy

A structured literature search was conducted to identify relevant studies on bilateral asymmetrical hip dislocation with acetabular fractures. The following databases were searched:

- PubMed;
- EMBASE;
- Scopus;
- Web of Science;
- Google Scholar (for gray literature and additional case reports).

The search was performed in December 2024, with no language restrictions. The search string used was as follows:

(“bilateral hip dislocation” OR “asymmetrical hip dislocation”) AND (“acetabular fracture” OR “hip trauma”) AND (“treatment” OR “management” OR “outcomes”).

The initial search yielded 69 articles, which were screened based on relevance, with particular focus on case reports and small case series detailing injury mechanisms, surgical management, postoperative rehabilitation, and long-term functional outcomes. The reference lists of selected articles were manually reviewed to identify any additional relevant reports.

#### 3.1.2. Organization of the Search and Selection Process

Two independent investigators (J.G. and U.D.) reviewed all 69 retrieved articles. After screening for relevance, 12 case reports and small case series were included in the final review. Articles that lacked detailed patient data, focused on pediatric cases, or described developmental hip dislocations were excluded. Disagreements in article selection were resolved through discussion with a third investigator (N.B.). The final selection of articles was qualitatively synthesized to provide insights into treatment strategies, complications, and outcomes.

#### 3.1.3. Limitations of the Literature Review Approach

Since this study is a narrative review, no formal PRISMA flowchart, systematic inclusion/exclusion criteria, or risk of bias assessment were applied. Instead, we aimed to provide a broad, expert-driven synthesis of available literature while maintaining transparency in the search strategy and study selection.

### 3.2. Epidemiology and Incidence

Traumatic hip dislocations are rare but significant orthopedic injuries, comprising approximately 2–5% of all joint dislocations. Within this spectrum, posterior dislocations constitute the majority (85–90%), whereas anterior dislocations account for only 10–15%. The rarity of bilateral hip dislocations (1% of all hip dislocations) reflects the immense mechanical forces required to simultaneously disrupt the integrity of both femoral heads and acetabular articulations [6].

A far more uncommon subset of these injuries is asymmetrical bilateral hip dislocations, where one femoral head is displaced anteriorly and the contralateral head is displaced posteriorly. These injuries represent an exceedingly rare subset, occurring in only 0.01–0.02% of all joint dislocations, a frequency so low that only approximately 100 cases have been reported globally, with fewer than 33 documented in English-language orthopedic literature [6].

The demographic distribution of these injuries is highly skewed toward young males (aged 20–40 years), a trend largely attributed to higher exposure to high-energy trauma mechanisms, particularly motor vehicle collisions (MVCs), high-velocity falls, and occupational injuries. Notably, the incidence of these injuries has increased in recent decades, a pattern correlated with rising vehicular speeds, increased urbanization, and an overall increase in high-energy trauma mechanisms.

### 3.3. Biomechanics and Mechanism of Injury

The hip joint’s inherent osseoligamentous stability necessitates a significant force vector misalignment to induce dislocation. The ball-and-socket articulation of the femoral head and acetabulum, reinforced by the iliofemoral, pubofemoral, and ischiofemoral ligaments, ensures that isolated dislocations require an exceedingly high-magnitude force to overcome capsuloligamentous constraints.

In the context of asymmetrical bilateral hip dislocations, these opposing injury vectors arise from differential kinetic energy transmission to each lower extremity. The predominant mechanisms of injury include the following:

1. Dashboard Injuries (Most Frequent Etiology, ~70%)
  ○ During high-speed frontal MVCs, axial loading through a flexed femur forces the ipsilateral hip into posterior dislocation, while the contralateral limb, extended and abducted at impact, undergoes anterior displacement due to excessive external rotation [3,7].
2. Falls from Height
  ○ Eccentric impact distribution at landing may cause one limb to land in hyperflexion (posterior dislocation) while the other remains in hyperextension (anterior dislocation), resulting in divergent femoral head displacements [8].
3. Pedestrian–Vehicle Trauma
  ○ Side-impact forces impose asymmetric rotational vectors on the pelvis, resulting in contralateral dislocation patterns depending on lower limb positioning [9].
4. Industrial Crush Injuries
  ○ Differential compressive loads on the lower extremities can exert asynchronous shearing forces, leading to simultaneous but opposing dislocations.

### 3.4. Associated Injuries and Pathophysiological Considerations

Due to the sheer magnitude of force required to produce an asymmetrical bilateral hip dislocation, these injuries rarely occur in isolation. Concomitant fractures, neurovascular injuries, and polytrauma sequelae are exceedingly common, warranting a meticulous and systematic trauma assessment.

#### 3.4.1. Acetabular Fractures (30–50%)

Acetabular involvement occurs primarily in posterior dislocations, given the posterior wall’s vulnerability to impaction during femoral head translation. In cases involving anterior dislocation, fractures tend to involve the anterior column or quadrilateral plate, particularly when high-velocity shearing forces are present. Fracture stability dictates management, with nonoperative strategies reserved for minimally displaced fragments, while surgical fixation is mandatory for unstable fractures (>20–25% posterior wall involvement, intra-articular fragments, or residual incongruence) [4].

#### 3.4.2. Femoral Head Fractures (15–30%)

The Pipkin classification delineates femoral head fracture patterns, with Type II-IV injuries carrying a significantly increased risk of avascular necrosis (AVN). The vascular supply to the femoral head, predominantly via the medial femoral circumflex artery, is highly susceptible to disruption, particularly in fractures with fragment displacement or delayed reduction (>6 h) [10,11].

#### 3.4.3. Pelvic Ring Injuries (10–20%)

Complex pelvic trauma, including pubic symphysis diastasis and sacroiliac instability, is frequently observed in high-energy pedestrian and fall-related injuries. These injuries compromise pelvic ring integrity, often necessitating stabilization via anterior plating or percutaneous iliosacral screw fixation [12].

#### 3.4.4. Neurological Injuries (10–15%)

Sciatic nerve involvement predominates in posterior dislocations, particularly when the femoral head impinges upon the sciatic notch. Peroneal branch dysfunction leads to foot drop, sensory deficits, and compromised dorsiflexion strength, while tibial branch impairment manifests as plantar flexion weakness. In anterior dislocations, femoral nerve dysfunction is possible, resulting in quadriceps weakness and sensory loss in the anteromedial thigh [13].

### 3.5. Imaging and Diagnostic Considerations

#### 3.5.1. Radiographic and Computed Tomographic Assessment

Anteroposterior pelvic radiographs remain the first-line modality, offering rapid characterization of dislocation type, femoral head translation, and acetabular integrity. However, radiographs alone often fail to detect intra-articular fractures, necessitating computed tomography (CT) for preoperative planning.

CT imaging is paramount in defining fracture morphology, identifying loose bodies, and assessing post-reduction congruity. Three-dimensional CT reconstructions further facilitate surgical approach selection, particularly in acetabular fractures requiring ORIF [13,14].

#### 3.5.2. MRI for AVN Risk Stratification

Magnetic resonance imaging (MRI) serves a critical role in AVN surveillance, particularly in cases with delayed reduction (>6 h), femoral head fractures, or multiple reduction attempts. Diffusion-weighted imaging (DWI) and contrast-enhanced sequences provide early detection of subclinical ischemia, informing long-term prognosis and potential need for joint-preserving interventions [15].

### 3.6. Management Strategies

#### 3.6.1. Principles of Reduction and Surgical Decision Making

The cornerstone of managing asymmetrical bilateral hip dislocations is the timely and precise reduction of the dislocated femoral heads, with the overarching goal of restoring joint congruity, minimizing articular cartilage damage, and preventing long-term complications such as avascular necrosis (AVN) and post-traumatic arthritis. In the setting of bilateral involvement, the approach to reduction and definitive management must be strategically planned, prioritizing closed reduction within the critical six-hour window whenever possible to mitigate ischemic insult to the femoral head.

Although closed reduction is successful in 85–90% of simple hip dislocations, failure rates increase significantly in cases involving concurrent acetabular fractures, femoral head fractures, soft-tissue interposition, or impaction injuries. Consequently, understanding the biomechanics of reduction, failure patterns, and the indications for surgical intervention is paramount.

#### 3.6.2. Closed Reduction Techniques: Biomechanical Considerations and Failure Mechanisms

- Reduction of Posterior Hip Dislocations: Allis and Stimson Maneuvers

For posterior dislocations, the Allis maneuver is the most widely employed technique, utilizing controlled axial traction, flexion, and rotational realignment to relocate the femoral head. The patient is positioned supine, with the hip and knee flexed to 90 degrees. The surgeon applies longitudinal traction along the femoral axis while simultaneously exerting gentle internal and external rotational forces to disengage the femoral head from the posterior acetabular rim. An assistant stabilizes the pelvis to counteract rotational displacement, ensuring optimal force transmission.

An alternative is the Stimson maneuver, particularly effective in cases where muscle spasm or posterior capsule impingement complicates standard reduction. The patient is positioned prone on an elevated surface with the affected limb hanging freely, allowing gravity-assisted reduction. The surgeon applies gradual downward traction at the knee while simultaneously internally rotating the hip, facilitating femoral head re-entry into the acetabulum [16].

- Reduction of Anterior Hip Dislocations: Captain Morgan and Reverse Bigelow Maneuvers

For anterior dislocations, the Captain Morgan technique provides direct mechanical leverage for controlled reduction. The patient remains supine, and the clinician places their knee beneath the affected limb while applying progressive downward pressure on the proximal thigh, coupled with controlled external rotation and flexion to guide the femoral head back into the acetabulum.

The Reverse Bigelow maneuver is employed when an anteriorly dislocated femoral head is locked against the anterior acetabular rim. Here, the surgeon applies traction and gentle extension, allowing the femoral head to clear the rim before executing gradual external rotation [7,16].

#### 3.6.3. Failure of Closed Reduction: Indications for Open Reduction and Internal Fixation (ORIF)

Closed reduction fails in 10–15% of cases, typically due to interposed soft tissue, osteochondral fragments, or acetabular impaction injuries. The presence of an irreducible femoral head, unstable post-reduction alignment, or intra-articular fragments mandates open reduction.

Surgical intervention is also required in cases of the following:

1. Acetabular fractures exceeding 25% of the weight-bearing dome;
2. Femoral head fractures (Pipkin Type II–IV) with fragment displacement >2 mm;
3. Marginal impaction of the posterior acetabular wall (gull-wing deformity);
4. Persistent joint instability despite attempted reduction.

#### 3.6.4. Surgical Approaches and Fixation Strategies

The choice of surgical approach is dictated by fracture pattern, femoral head involvement, and acetabular integrity.

1. Kocher–Langenbeck Approach for Posterior Wall and Column Fractures

The Kocher–Langenbeck approach remains the gold standard for posterior acetabular wall and column fractures. This posterior exposure facilitates direct access to impacted fracture fragments, allowing accurate reduction and rigid internal fixation using reconstruction plates. However, due to its proximity to the sciatic nerve, meticulous dissection is required to prevent iatrogenic neuropathy [17].

2. Ilioinguinal Approach for Anterior Column and Quadrilateral Plate Fractures

For anterior column involvement, the ilioinguinal approach provides optimal exposure to the anterior acetabulum, quadrilateral plate, and pelvic brim. This approach is preferred when ORIF is indicated for anterior column fractures or in cases requiring stabilization of both columns [18].

3. Smith–Petersen Approach for Femoral Head ORIF

Pipkin fractures involving the superior weight-bearing dome of the femoral head necessitate ORIF using a Smith–Petersen approach, which provides direct access to the femoral head while minimizing soft-tissue disruption [19].

### 3.7. Postoperative Protocols and Rehabilitation

The postoperative management of asymmetrical bilateral hip dislocations is tailored to fracture stability, risk of AVN, and individual patient factors. A structured, phase-based rehabilitation program is essential to restore joint mobility, prevent stiffness, and optimize long-term function [7].

#### 3.7.1. Phase 1: Early Postoperative Period (Weeks 1–6)

The initial phase focuses on protected weight-bearing and joint stability. In cases of isolated dislocations without fracture, weight-bearing is typically restricted for 2–4 weeks, allowing capsular healing. However, in fracture-associated dislocations, a strict 6- to 12-week non-weight-bearing protocol is mandated to prevent displacement and allow bony consolidation.

Deep vein thrombosis (DVT) prophylaxis using low-molecular-weight heparin (LMWH) or direct oral anticoagulants (DOACs) is essential due to prolonged immobility and venous stasis.

#### 3.7.2. Phase 2: Progressive Mobilization (Weeks 6–12)

Once radiographic evidence of fracture healing is confirmed, patients transition to partial weight-bearing with crutches, advancing to full weight-bearing by 10–12 weeks. At this stage, strengthening exercises targeting gluteal and quadriceps function are introduced, alongside closed-chain proprioceptive training to re-establish neuromuscular control.

MRI at six weeks post-injury is crucial for early detection of AVN, particularly in cases with delayed reduction or femoral head involvement.

#### 3.7.3. Phase 3: Functional Restoration and Long-Term Surveillance (Beyond 12 Weeks)

The final rehabilitation phase focuses on functional restoration, incorporating advanced neuromuscular re-education, gait retraining, and progressive loading exercises. Patients recovering from ORIF or complex dislocations require serial MRI scans every 6 to 12 months for two years to monitor for AVN progression.

Long-term surveillance is essential due to high rates of post-traumatic osteoarthritis (20–50%), necessitating early intervention strategies to delay the need for total hip arthroplasty (THA).

## 4. Discussion

Bilateral asymmetrical hip dislocations remain one of the rarest presentations in orthopedic trauma, with limited cases documented in the literature. The injury pattern seen in our patient—a right posterior hip dislocation with an associated posterior wall acetabular fracture and a left anterior-inferior (obturator-type) dislocation—aligns with the majority of previously reported cases that describe dashboard-type trauma as the primary mechanism [1,2,3,13,20]. However, a key distinction in our case is the absence of concurrent femoral head fractures, which are reported in 15–30% of cases, particularly in those with delayed reduction.

### 4.1. Comparison of Reported Mechanisms and Associated Injuries

A review of previously documented cases confirms motor vehicle collisions (MVCs) as the predominant cause of bilateral asymmetrical hip dislocations, with dashboard injuries accounting for over 70% of cases [13]. The mechanism of injury in our patient follows this established pattern, wherein axial loading of the flexed hip produced posterior dislocation on the right, while hyperextension, abduction, and external rotation forces induced anterior dislocation on the left. This mechanism closely mirrors findings by Alshammari et al., who reported a nearly identical injury pattern in an MVC trauma case [3].

Notably, our case differs in the pattern of associated injuries. While up to 50% of posterior dislocations are associated with acetabular fractures, our patient sustained a posterior wall acetabular fracture without additional pelvic ring disruption. This contrasts with cases such as those reported by Hamilton et al. and other relevant articles [10,11,12,21], where simultaneous pelvic fractures or femoral neck fractures were present. The absence of femoral head fractures in our patient suggests that the impact vector predominantly affected the acetabular rim rather than inducing direct shear stress on the femoral head, a critical factor in reducing the risk of Pipkin-type fractures and subsequent avascular necrosis (AVN).

### 4.2. Timing of Reduction and AVN Risk

The timing of hip reduction remains the most critical determinant of long-term outcomes, particularly in minimizing the risk of AVN. Avascular necrosis of the femoral head occurs in 4.8% of cases when reduction is achieved within six hours but rises to 58.8% in cases where reduction is delayed beyond this threshold. In our case, reduction was performed within two hours post-injury, significantly lowering the risk of AVN development [22].

A comparative analysis of previous studies indicates variability in the timing of reduction and its impact on femoral head viability.

- The timing of hip reduction is a critical determinant in minimizing the risk of avascular necrosis (AVN) of the femoral head. A meta-analysis by Ahmed et al. (2017) found that traumatic hip dislocations reduced within six hours had a significantly lower incidence of AVN compared to those reduced after six hours [23].
- Multiple attempts at closed reduction of hip dislocations have been associated with an increased risk of avascular necrosis (AVN) of the femoral head. According to a case report by Kim et al., “repeated or forceful closed reduction of irreducible femoral head fracture-dislocation injuries may result in iatrogenic femoral neck fractures”. The report recommends that “in an irreducible hip dislocation, without further attempting for closed reduction, an immediate open reduction is recommended” to prevent complications such as AVN [24].

Given that our patient underwent successful closed reduction without the need for repeated manipulations, followed by immediate radiographic confirmation of concentric reduction, the likelihood of developing osteonecrosis remains relatively low, reinforcing the importance of early intervention.

### 4.3. Surgical Management: Case-Specific Considerations vs. Literature Trends

The decision to proceed with open reduction and internal fixation (ORIF) of the posterior wall acetabular fracture was guided by well-established biomechanical criteria. Literature suggests that acetabular fractures involving >25% of the posterior wall, leading to joint instability, are clear indications for ORIF. In our case, post-reduction imaging confirmed persistent instability, necessitating definitive stabilization via the Kocher–Langenbeck approach.

A review of previously reported surgical cases highlights a divergence in surgical strategies based on the fracture pattern:

- Caviglia et al. utilized percutaneous screw fixation for select posterior wall fractures, demonstrating good outcomes in minimally displaced fragments [25].
- Tosounidis et al. emphasized the Kocher–Langenbeck approach as the gold standard for posterior acetabular fractures, particularly in cases of comminution or articular step-off > 2 mm [17].
- In anterior dislocations with associated anterior column fractures, approaches such as ilioinguinal or modified Stoppa techniques are often preferred [18,19].

Given our patient’s isolated posterior wall fracture with no additional pelvic ring instability, the Kocher–Langenbeck approach with 3.5 mm reconstruction plate fixation was the most appropriate choice. Immediate postoperative stability was confirmed intraoperatively.

### 4.4. Rehabilitation and Functional Outcomes in Context

Long-term outcomes following asymmetrical bilateral hip dislocations depend on multiple factors, including the presence of associated fractures, timing of reduction, and the implementation of an appropriate post-injury rehabilitation protocol. Literature suggests that patients who achieve full concentric reduction within six hours, undergo early mobilization, and follow a structured rehabilitation program tend to have favorable long-term function and lower rates of complications such as avascular necrosis (AVN) and post-traumatic osteoarthritis [13].

Our patient was initiated on a structured, phased rehabilitation program, which included the following:

1. Toe-touch weight-bearing for six weeks, progressively advancing to full weight-bearing by 12 weeks to allow for adequate fracture healing and prevent early joint overload.
2. DVT prophylaxis with LMWH to minimize thromboembolic risk, particularly in the early non-weight-bearing phase.
3. Serial imaging follow-ups at three and six months to assess fracture healing, joint congruity, and signs of AVN.

Recent studies have highlighted the role of adjunctive rehabilitative modalities in optimizing post-surgical recovery. Mariconda et al. (2020) demonstrated that a combination of therapeutic exercise and radiofrequency ablation can significantly reduce hip pain and enhance joint function in patients with hip osteoarthritis, suggesting that similar approaches could be beneficial in post-traumatic hip rehabilitation [26]. Additionally, Notarnicola et al. (2023) reported that extracorporeal shock wave therapy (ESWT), when combined with structured therapeutic exercises, improved pain relief and functional outcomes in patients with hip pain syndromes [27]. These findings suggest that incorporating targeted neuromuscular training, pain management strategies such as ESWT or radiofrequency ablation, and progressive weight-bearing protocols may further enhance recovery in patients with complex hip injuries.

At six months, our patient had achieved full functional recovery, was pain-free, and demonstrated complete range of motion, consistent with previous studies indicating that early surgical stabilization and comprehensive rehabilitation lead to optimal long-term outcomes. Future research should explore the potential role of adjunctive rehabilitation strategies in reducing pain, preventing complications, and enhancing functional recovery following complex hip trauma.

### 4.5. Prognostic Implications and Areas for Further Research

The prognosis of bilateral asymmetrical hip dislocations is largely dictated by the extent of associated injuries, the timing of reduction, and the stability of post-reduction alignment. Our case exemplifies optimal management principles, highlighting the importance of the following:

- Expedited closed reduction to minimize AVN risk.
- Early surgical stabilization in cases of instability.
- Structured rehabilitation to optimize functional recovery.

However, gaps remain in the long-term surveillance of these patients, particularly regarding the following:

- The true incidence of post-traumatic osteoarthritis beyond five years.
- The impact of residual microinstability in cases where closed reduction is achieved without ORIF.
- The role of biological adjuvants (bisphosphonates, hyperbaric oxygen) in mitigating AVN progression.

### 4.6. Limitations of Our Study

While this case provides valuable insights into the management and outcomes of bilateral asymmetrical hip dislocations, several limitations should be acknowledged. As a single case report, its findings may not be universally generalizable, given the variability in injury patterns, patient demographics, and treatment protocols across different trauma centers. Additionally, long-term functional outcomes beyond six months remain unknown, as late complications such as post-traumatic osteoarthritis or avascular necrosis (AVN) may develop years post-injury.

Another limitation is the lack of comparative treatment approaches, as this case was managed with early closed reduction, definitive surgical fixation, and structured rehabilitation. While this approach aligns with best practices, further research is needed to evaluate whether alternative fixation strategies, earlier mobilization protocols, or biological therapies could enhance recovery or mitigate long-term complications.

Moreover, the rarity of bilateral asymmetrical hip dislocations presents a challenge in establishing large-scale prospective studies, limiting the ability to derive evidence-based guidelines on optimal reduction techniques, surgical decision making, and rehabilitation strategies. Future research should focus on multi-center data collection, long-term functional analysis, and advancements in AVN prevention to further refine treatment paradigms for this rare but complex injury.

## 5. Conclusions

Bilateral asymmetrical hip dislocations are exceptionally rare, requiring rapid recognition, prompt reduction, and individualized treatment strategies. Our case of a right posterior dislocation with a posterior wall acetabular fracture and a left anterior-inferior dislocation, sustained in a high-energy MVC, reinforces the importance of early intervention in preventing complications such as avascular necrosis (AVN) and post-traumatic instability.

The literature highlights that early reduction within six hours significantly lowers AVN risk, while persistent instability necessitates timely surgical fixation. Our patient’s successful closed reduction, definitive ORIF via the Kocher–Langenbeck approach, and structured rehabilitation led to full functional recovery with no radiographic complications at six months.

Despite well-established treatment principles, long-term data on functional outcomes, post-traumatic osteoarthritis, and AVN mitigation remain limited. This case underscores the need for continued research into optimizing rehabilitation and refining surgical indications. Ultimately, expedited intervention and evidence-based management remain key to achieving optimal outcomes in these complex injuries.

## Figures and Tables

**Table 1:** Timeline of events.

Event	Timeframe
High-speed MVC occurs	Day 0
Patient arrives at trauma center	1 h post-injury
Initial evaluation, imaging, and diagnosis	1–2 h post-injury
Closed reduction performed under anesthesia	2 h post-injury
Post-reduction imaging confirms joint congruity but unstable acetabular fracture	3 h post-injury
Open reduction and internal fixation (ORIF) performed	Day 3
Discharged with rehabilitation protocol	2 weeks post-op
Progressed to partial weight-bearing	6 weeks post-op
Full weight-bearing with complete ROM	12 weeks post-op
Final follow-up—no AVN or osteoarthritis	6 months post-op

## Data Availability

All data are available in references.

## Abbreviations

The following abbreviations are used in this manuscript:

ORIF	Open Reduction and Internal Fixation
AVN	Avascular Necrosis
MVC	Motor Vehicle Collision
ATLS	Advanced Trauma Life Support
AP	Anteroposterior
MSCT	Multislice Computed Tomography
3D	Three-Dimensional
DVT	Deep Vein Thrombosis
LMWH	Low-Molecular-Weight Heparin
HHS	Harris Hip Score
TUG	Timed Up and Go
BBS	Berg Balance Scale
CT	Computed Tomography
MRI	Magnetic Resonance Imaging
DWI	Diffusion-Weighted Imaging
THA	Total Hip Arthroplasty

## References

[1] Chung K.J., Eom S.W., Noh K.C., Kim H.K., Hwang J.H., Yoon H.S., Yoo J.H. (2009). Bilateral Traumatic Anterior Dislocation of the Hip with an Unstable Lumbar Burst Fracture. Clin. Orthop. Surg..

[2] Meena U.K., Joshi N., Behera P., Meena B.P., Jain S. (2021). Simultaneous asymmetrical bilateral hip dislocations—A report of three cases with different presentations and management. J. Clin. Orthop. Trauma.

[3] Alshammari A., Alanazi B., Almogbil I., Alfayez S.M. (2018). Asymmetric bilateral traumatic hip dislocation: A case report. Ann. Med. Surg..

[4] Alonso J.E., Volgas D.A., Giordano V., Stannard J.P. (2000). A review of the treatment of hip dislocations associated with acetabular fractures. Clin. Orthop. Relat. Res..

[5] Milenkovic S., Mitkovic M., Mitkovic M. (2022). Avascular necrosis of the femoral head after traumatic posterior hip dislocation with and without acetabular fracture. Eur. J. Trauma Emerg. Surg..

[6] Alnaser A.A.M.A., Abd-Elmaged H.M.A., Mohammed F.E.A., Abd Allah R.A.A.A., Mohamed Ahmed Hussien M.A. (2022). A bilateral asymmetrical hip dislocation: A rare case report. Clin. Case Rep..

[7] Dawson-Amoah K., Raszewski J., Duplantier N., Waddell B.S. (2018). Dislocation of the Hip: A Review of Types, Causes, and Treatment. Ochsner. J..

[8] Kohandel O., Mirhoseini M.S., Mirrahimi S.S. (2024). Bilateral asymmetrical hip dislocations after falling from height: A case report and literature review. J. Orthop. Spine Trauma.

[9] Qin W., Fang Y. (2021). Traumatic asymmetrical bilateral hip dislocation: A rare case report. Jt. Dis. Relat. Surg..

[10] Mortazavi S.J., Mazzochy H., Ghasemi M., Khan F. (2020). Asymmetric Bilateral Hip Fracture-Dislocation: A Case Report and Literature Review. J. Orthop. Spine Trauma.

[11] Değirmenci E., Kaya Y.E., Özturan K.E. (2018). Asymmetric bilateral hip dislocations and unilateral femoral head fracture: A CASE report. Trauma Case Rep..

[12] Shermetaro J., Gard J., Brossy K. (2022). Asymmetric Bilateral Hip Dislocations With an Associated Unstable Pelvic Ring Injury: A Case Report. Cureus.

[13] Buckwalter J., Westerlind B., Karam M. (2015). Asymmetric Bilateral Hip Dislocations: A Case Report and Historical Review of the Literature. Iowa Orthop. J..

[14] Park H.G., Yi H.S., Han K.H. (2018). Bilateral Asymmetric Traumatic Dislocation of the Hip Joint. J. Trauma Inj..

[15] Väänänen M., Tervonen O., Nevalainen M.T. (2021). Magnetic resonance imaging of avascular necrosis of the femoral head: Predictive findings of total hip arthroplasty. Acta Radiol. Open.

[16] Waddell B.S., Mohamed S., Glomset J.T., Meyer M.S. (2016). A Detailed Review of Hip Reduction Maneuvers: A Focus on Physician Safety and Introduction of the Waddell Technique. Orthop. Rev..

[17] Tosounidis T.H., Giannoudis V.P., Kanakaris N.K., Giannoudis P.V. (2018). The Kocher-Langenbeck Approach:State of the Art. JBJS Essent. Surg. Tech..

[18] Tosounidis T.H., Giannoudis V.P., Kanakaris N.K., Giannoudis P.V. (2018). The Ilioinguinal Approach: State of the Art. JBJS Essent. Surg. Tech..

[19] Ricci W.M., McAndrew C.M., Miller A.N., Avery M.C. (2018). Open Reduction and Internal Fixation of the Femoral Head via the Smith-Petersen Approach. J. Orthop. Trauma.

[20] Nandi R., Das P., Nandi S.N. (2021). A Case Report of Bilateral Asymmetrical Traumatic Hip Dislocation—A Rare Presentation. J. Orthop. Case Rep..

[21] Hamilton D.A., Wright R.D., Moghadamian E.S., Bruce B.T., Selby J.B. (2012). Bilateral asymmetric hip dislocation: A case series and literature review of a rare injury pattern. J. Trauma Acute Care Surg..

[22] Kellam P., Ostrum R.F. (2016). Systematic Review and Meta-Analysis of Avascular Necrosis and Posttraumatic Arthritis After Traumatic Hip Dislocation. J. Orthop. Trauma.

[23] Ahmed G., Shiraz S., Riaz M., Ibrahim T. (2017). Late versus early reduction in traumatic hip dislocations: A meta-analysis. Eur. J. Orthop. Surg. Traumatol..

[24] Kim T.S., Oh C.W., Kim J.W., Park K.H. (2018). An Irreducible Hip Dislocation with Femoral Head Fracture. J. Trauma Inj..

[25] Caviglia H., Mejail A., Landro M.E., Vatani N. (2018). Percutaneous fixation of acetabular fractures. EFORT Open Rev..

[26] Mariconda C., Megna M., Farì G., Bianchi F.P., Puntillo F., Correggia C., Fiore P. (2020). Therapeutic exercise and radiofrequency in the rehabilitation project for hip osteoarthritis pain. Eur. J. Phys. Rehabil. Med..

[27] Notarnicola A., Ladisa I., Lanzilotta P., Bizzoca D., Covelli I., Bianchi F.P., Maccagnano G., Farì G., Moretti B. (2023). Shock Waves and Therapeutic Exercise in Greater Trochanteric Pain Syndrome: A Prospective Randomized Clinical Trial with Cross-Over. J. Pers. Med..

